# Stigma during monkeypox outbreak

**DOI:** 10.3389/fpubh.2022.1023519

**Published:** 2022-09-20

**Authors:** Ranjit Sah, Aroop Mohanty, Abdullah Reda, Bijaya Kumar Padhi, Alfonso J. Rodriguez-Morales

**Affiliations:** ^1^Department of Microbiology, Institute of Medicine, Tribhuvan University Teaching Hospital, Kathmandu, Nepal; ^2^Faculty of Medicine, Harvard Medical School, Boston, MA, United States; ^3^All India Institute of Medical Sciences, Gorakhpur, India; ^4^Faculty of Medicine, Al-Azhar University, Cairo, Egypt; ^5^Department of Community Medicine and School of Public Health, Postgraduate Institute of Medical Education and Research, Chandigarh, India; ^6^Grupo de Investigación Biomedicina, Faculty of Medicine, Fundacion Universitaria Autonoma de las Americas, Pereira, Colombia; ^7^Clinical Epidemiology and Biostatistics, Universidad Cientifica del Sur, Lima, Peru

**Keywords:** stigma, monkeypox, outbreak, COVID-19, discrimination, healthcare worker

Monkeypox is an emerging viral zoonotic disease that is endemic in Central and West Africa. The first cases outside the African continent were detected in the United States of America (USA) in 2003 following the importation of infected animals. On July 23, 2022, the World Health Organization (WHO) declared monkeypox a Public Health Emergency of International Concern (PHIEC), following the rapid spreading into multiple continents in short time (1). Since July 6, 2022, 12,989 confirmed monkeypox cases have been detected, showing a more than 200% increase. The total number of cases has gone beyond 21,000 cases and has spread to 75 countries involving all six WHO regions (www.who.int) up to July 30, 2022. We need to increase the awareness about disease transmission, signs and symptoms, and how to prevent ourselves from this emerging infection. Lessons from research on past infectious disease outbreaks have taught us that stigma will pose a significant hurdle to health and wellness during any pandemic.

This problem of stigma needs to be identified in the current monkeypox pandemic. A stigma is a negative attitude about a person's mental, physical or social features or group of people. It is considered a type of social disapproval when the affected person thinks that society will not accept him/her due to this condition. Such social stigma in case of an outbreak can lead to many adverse effects, especially in low- and middle-income countries. That may mean people are being labeled, stereotyped, discriminated against, treated separately or are experiencing a loss of status given a perceived link with diseases (2). Stigma makes the people to hide the disease which boost the untrace spreading of the virus and making the outbreak worse. It has been seen in many infectious diseases, including HIV/AIDS, H1N1 and more recently in COVID-19, mainly due to false news published about a new condition and the fear of death when that new disease is associated with significant morbidity and fatality rates. In general, a new disease is always associated with many unknowns, and we are afraid of those unknowns, and thus, fear can be easily associated with them. New diseases transmitted *via* air and droplet are invariably associated with high social stigma, and COVID-19 is a perfect example of that. Healthcare workers and patients who survived COVID-19 faced substantial social stigma and discrimination worldwide. It created unprecedented panic in people's minds, with several socially stigmatized. Such discrimination resulted in affected people hiding their illness, prevented them from visiting hospitals and ultimately led to certain psychological problems such as anxiety and depression and grave complications from the diseases due to lack of proper health care (3).

Such social stigma has now started appearing in monkeypox patients as the first confirmed case in Thailand could not be traced for nearly 5 days. He was a 27 years old Nigerian male who tested positive on July 18, 2022, in Phuket, Thailand, after which he went on the run, turned off his phone and failed to respond to messages from police and healthcare workers. After a countrywide chase across Thailand, his phone signal was detected in a northeastern province bordering Cambodia. Then Cambodian police captured him at Phnom Penh guest house and sent him to the Khmer–Soviet Friendship Hospital for medical care. The story of this Nigerian guy was like a movie where two country police were together trying to tract a single monkeypox-infected patient just like they were searching for some international mafia/terrorist. During the whole episode, he infected many others, all of whom are being screened. He had visited two entertainment venues and had unprotected sex with women. As a result, 142 people are now being screened for the monkeypox virus. Due to stigma, people avoid admitting infection or signs/symptoms and unknowingly spread the disease to others. This episode demonstrates the rising stigma associated with monkeypox and the difficulty in fighting the spread of the disease (4). The question is, then, how to combat this stigma associated with monkeypox?

Addressing stigma is fundamental in healthcare sector. Numerous studies during the COVID-19 pandemic have highlighted stigma and discrimination among healthcare workers. Lessons from COVID-19, which showed healthcare workers are vulnerable to such kind of emerging infections (5–7). Healthcare workers specifically belonging to LGBTQ+ community who get exposed to monkeypox virus can also hide their diseases status due to the stigma in the initial phase of the diseases and poses a threat to transmit the infection in the hospital setting during the working hours. Therefore, healthcare authorities should adopt educational campaigns to relieve this stigma and urge infected/suspected cases to report the infection and properly isolate themselves until proper care is provided. To reduce the social stigma associated with monkeypox, the Center for Disease Control and Prevention (CDC) has framed specific recommendations, including educating the general public about the disease as a legitimate public health concern (www.cdc.gov). It must be clear to people that everyone can acquire monkeypox, regardless of gender identity or sexual orientation, or age. Proper emphasis regarding the different modes of transmission, including direct contact with the infectious rash, respiratory secretions during face-to-face contact and touching infected objects, is also being given. We also should not stigmatize the gay and bisexual community (or in general LGBTIQ+ one) by holding them responsible for spreading the disease. They should be reached using targeted channels like specific websites, dating apps, or media programs, with a holistic approach. Reliable sources such as the CDC and WHO should be sought to obtain correct information about the disease. Any sign or symptom of the disease should be reported at the earliest, knowing that it is associated with a good prognosis in most cases. Besides all of the above, effective and impactful communication about monkeypox is critical to combat the disease and avoid fueling fear and stigma. It includes using friendly words with the patients, promoting the general population to participate in the spread of accurate scientific news and especially the social influencers whose voices have an impact (5–7). It is critically important to develop culturally acceptable health promoting messages that provide accurate and timely information on monkeypox its symptoms and prevention strategies. This certainly enable communities to fight against the disease. That ultimately helps create an environment where the disease and its effects can be discussed openly, honestly and effectively, finally helping in the prevention and control of the disease and the outbreak. Studies should explore how the Monkeypox stigma leads to differential diagnosis of the disease and its health outcomes including mental hygiene.

## Author contributions

RS: draw and draft the manuscript. BP, AM, AR, and AR-M: review the literature, finalize, and edit the manuscript. All authors have read and approve for the final manuscript.

## Conflict of interest

The authors declare that the research was conducted in the absence of any commercial or financial relationships that could be construed as a potential conflict of interest. The handling editor SB declared a shared affiliation with the author AM at the time of review.

## Publisher's note

All claims expressed in this article are solely those of the authors and do not necessarily represent those of their affiliated organizations, or those of the publisher, the editors and the reviewers. Any product that may be evaluated in this article, or claim that may be made by its manufacturer, is not guaranteed or endorsed by the publisher.
